# Discrepancy of flowering time between genetically close sublineages of *Aegilops umbellulata* Zhuk.

**DOI:** 10.1038/s41598-024-57935-w

**Published:** 2024-03-28

**Authors:** In Son, Nozomi Kasazumi, Moeko Okada, Shigeo Takumi, Kentaro Yoshida

**Affiliations:** 1 Graduate School of Agricultural Science, Kobe University, Kobe, Japan; 2 Graduate School of Science and Technology, Niigata University, Niigata, Japan; 3 Graduate School of Agriculture, Kyoto University, Kyoto, Japan

**Keywords:** *Aegilops umbellulata*, RNA sequencing, Genetic diversity, Lineage differentiation, Latitudinal clines, Plant genetics, Genetic variation

## Abstract

*Aegilops umbellulata* Zhuk., a wild diploid wheat-related species, has been used as a genetic resource for several important agronomic traits. However, its genetic variations have not been comprehensively studied. We sequenced RNA from 114 accessions of *Ae. umbellulata* to evaluate DNA polymorphisms and phenotypic variations. Bayesian clustering and phylogenetic analysis based on SNPs detected by RNA sequencing revealed two divergent lineages, UmbL1 and UmbL2. The main differences between them were in the sizes of spikes and spikelets, and culm diameter. UmbL1 is divided into two sublineages, UmbL1e and UmbL1w. These genetic differences corresponded to geographic distributions. UmbL1e, UmbL1w, and UmbL2 are found in Turkey, Iran/Iraq, and Greece, respectively. Although UmbL1e and UmbL1w were genetically similar, flowering time and other morphological traits were more distinct between these sublineages than those between the lineages. This discrepancy can be explained by the latitudinal and longitudinal differences in habitats. Specifically, latitudinal clines of flowering time were clearly observed in *Ae. umbellulata*, strongly correlated with solar radiation in the winter season. This observation implies that latitudinal differences are a factor in differences in the flowering times of *Ae. umbellulata*. Differences in flowering time could influence other morphological differences and promote genetic divergence between sublineages.

## Introduction

In plants, divergence in phenotypic traits within species is often linked to differences in habitat, such as temperature, precipitation, radiation, and interactors, including insects and pathogens. Environmentally adaptive traits tend to dominate over non-adaptive traits in local populations. Traits that are sensitive to environmental changes, such as flowering, can be optimized for local habitats by natural selection^1,2^. Differences in the days to flowering could restrict gene flow between populations, even at the borders of their habitats, thereby enhancing genetic divergence between populations. Divergence in flowering time serves as pre-mating isolation for speciation^1–4^. An association between differences in flowering time and population structure has been reported for *Brachypodium distachyon*^5^. However, the flowering period is not always associated with population structure. In *Arabidopsis thaliana*, variation in flowering time is independent of population structure^6^.

Photoperiod and temperature are key factors affecting the onset of flowering in temperate plant species^7^. Plants that require vernalization for flowering especially need to be exposed to low temperatures for a period of time during the winter. Genes involved in the vernalization, photoperiod, and circadian clock pathways regulate the transition from the vegetative to the reproductive stage^8^. Annual and diurnal variations in photoperiod and temperature vary with latitude. Polymorphisms in genes involved in vernalization or photoperiod are thought to generate latitudinal clines. In fact, for several plants, latitudinal clines of flowering time are observed, although the strength of clinal variation depends on sampling scales^9^. In *Arabidopsis thaliana*, the MADS-box transcription factor *FLC* is associated with vernalization and functions as a floral repressor of the florigen gene *FT* with two major alleles, *FLC*A and *FLC*B. The distribution of these alleles, in the presence of the functional allele *FRIGIDA* (*FRI*), is associated with the latitudinal cline of flowering time in Eurasia and North America^10–12^. The polymorphisms in the *Ppd-D1* gene, which is involved in photoperiod sensitivity, explain the latitudinal cline of flowering time in common wheat^13,14^. Differences in flowering time were also associated with other phenotypic traits^6,15–18^. In maize, polymorphisms in the *dwarf8* gene involved with plant height, are associated with flowering time in elite European inbred lines^16^. In *A. thaliana*, a latitudinal cline was detected in the covariance between primary dormancy, growth rate, and flowering time^6^.

Diversification of flowering time also exists in wild wheat relatives. Significant longitudinal and latitudinal clines in days to flowering were observed in *Aegilops tauschii* which consists of three divergent lineages: TauL1, TauL2 and TauL3^13,18,19^. In the Transcaucasus-Middle East, the flowering time in *Ae. tauschii* is positively correlated with latitude. Photoperiod may be a factor that forms a latitudinal cline. However, in Asia, flowering time variation shows a longitudinal cline. The sublineage TauL1b has colonized eastern Eurasia, ranging from Afghanistan to China. Early flowering of TauL1b has been linked to winter temperature^13,18^.

*Aegilops umbellulata* Zhuk is a diploid wild wheat relative with the U genome, distributed throughout Greece and the Middle East. *Ae. umbellulata* has been used as a genetic resource for disease resistance in common wheat^20–24^. The distribution of *Ae. umbellulata* overlapped with tetraploid wheat and einkorn wheat, whereas *Ae. umbellulata* did not contribute to the establishment of hexaploid wheat. An artificial cross between the tetraploid wheat *Triticum turgidum* ssp. *durum* cv. Langdon and *Ae. umbellulata* generated germinable triploid seeds (ABU genome)^25,26^. However, most hybrids were sterile and did not produce hexaploid seeds. The frequency of genome doubling in ABU hybrids was lower than that in the triploid hybrids of Langdon and *Ae. tauschii* (ABD genome), some combinations of which form unreduced gametes and produce hexaploid seeds^25^. Analysis of RNA-sequence based single nucleotide polymorphisms (SNPs) in 12 accessions of *Ae. umbellulata* showed no clear lineage differentiation^27^. Rarer allele frequency is predominant in *Ae. umbellulata*. Polymorphic patterns of *Ae. umbellulata* are distinct from those of *Ae. tauchii*. However, the number of accessions tested in these studies was too small to capture the population structure in *Ae. umbellulata*. In the present study, we aimed to reveal the population structure and phenotypic trait variations in *Ae. umbellulata*, we analyzed nucleotide polymorphisms in 114 accessions of *Ae. umbellulata* based on SNPs estimated by RNA sequencing (RNA-seq) of young seedlings and evaluated their phenotypic traits in multiple seasons.

## Results

### Construction of transcript sequences of *Ae. umbellulata*

**Figure 1:**
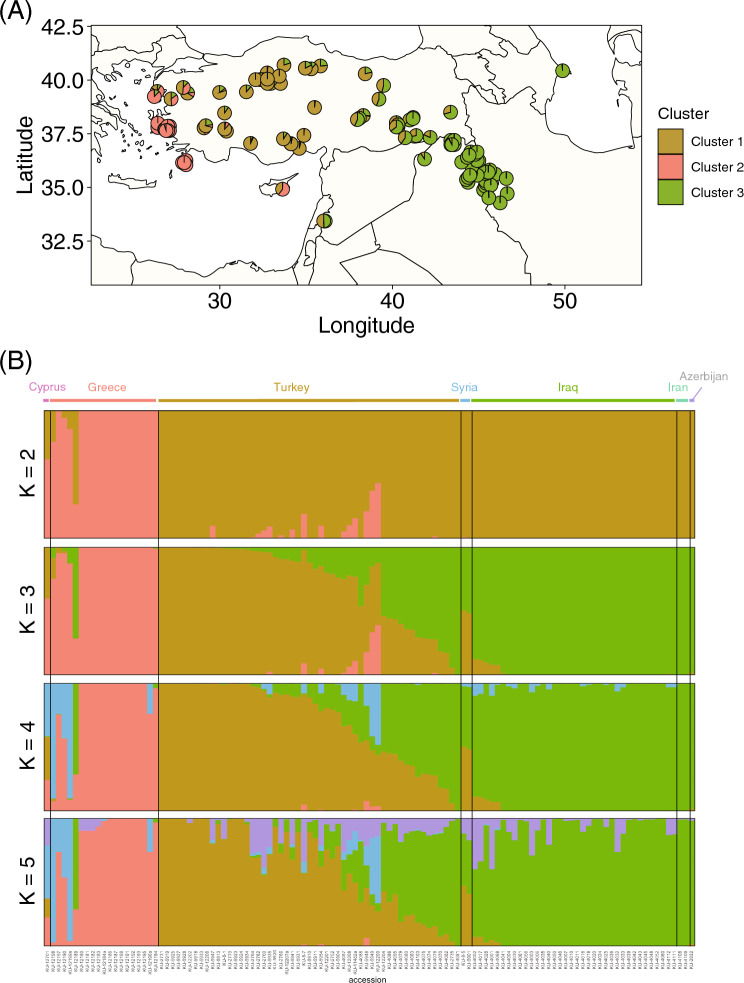
Population structure of the 114 accessions of *Aegilops umbellulata*. (**A**) Geographic distribution of the 114 *Ae. umbellulata* accessions, based on map dataset sourced from R package “ggplot2" ver. 3.5.0 in R software ver. 4.3.1. The color coding of the pie chart corresponds to the STRUCTURE results at K = 3 in (**B**) below. (**B**) Proportion of membership of the 114 accessions for K = 2 to 5, as calculated by STRUCTURE software based on SNPs that were called from the alignments of RNA-seq reads to the reference-based transcripts. Multiple runs for each K were concatenated using CLUMPP. Each accession, represented as a vertical line is partitioned into colored segments whose length is proportional to the individual's probability of assignment (Q-value) to the Kth cluster.

The National Bioresource Project-Wheat, Japan maintained the germplasm of *Ae. umbellulata*. The 114 accessions were randomly selected from the collection (Fig. 1A and Supplementary Table S1). Triticeae, including *Ae. umbellulata* have a large genome, and over 80% of the genome was composed of repeats derived from transposable elements^28,29^. Due to the difficulty of identifying high-confidence SNPs in transposable elements, we used RNA-seq of 3′-UTR for 114 accessions of *Ae. umbellulata* to estimate intraspecific DNA variation. The RNA-seq reads were aligned to two sets of transcript sequences. One is de novo assembly of transcripts that had been previously developed from 300 bp paired-end RNA-seq reads^27^. Another set of transcript sequences was reconstructed by substituting the nucleotides of *T. aestivum* cv. Chinese Spring (CS) D genome with *Ae. umbellulata* nucleotides at the SNP sites of high-confidence transcripts of CS^28^. Since *K*-mer based de novo assembly of RNA-seq short reads potentially generates chimeric transcripts, we also used the transcripts from CS D-genome that were developed based on whole-chromosome pseudomolecules. Based on the SNPs between *Ae. umbellulata* and wheat CS D-genomes, the nucleotides of the CS D-genome were replaced with those of *Ae. umbellulata*. To improve the SNP detection rate, these alignments and replacements were repeated 16 times, using the approach for improving alignments developed by Paape et al. 2018^30^. The number of detected SNPs decreased from 819,290 in the 1st repeat to 43 in the 13th repeat. Since the number of detected SNPs was almost constant after the 13th repeat (Supplementary Table S2), the deduced transcripts at the 13th repeat were used as a reference for the alignments and are referred to here as reference-based transcripts.

### Population structure in the 114 accessions of *Ae. umbellulata*

RNA-seq of the 114 accessions of *Ae. umbellulata* generated 367–1986 million reads per accession. The average number of sequencing reads per accession was 964 million. After quality control, filtered reads were aligned to the two sets of transcripts (Supplemental Table S3). Since *Ae. umbellulata* is selfing species, heterozygous SNPs are expected to be rare, and only homozygous SNPs were called. When the de novo assembled transcripts were used as a reference for alignments and SNP calling, 214–1522 million reads per accession were aligned, and 4455–21,236 SNPs per accession were called. A total of 202,692 SNP loci were identified. In contrast, when reference-based transcripts were used as a reference, 160–1059 million reads were aligned and 1393–10,996 SNPs were called. A total of 96,403 SNP loci were identified. The sample coverage per locus was higher in the SNP set based on de novo assembled transcripts than in the SNP set derived from reference-based transcripts (Supplemental Fig. S1). For the downstream analyses, we used biallelic and homozygous SNP loci with 100% sample coverage. Finally, the number of SNP loci selected based on de novo assembled transcripts and reference-based transcripts were 6286 and 4459, respectively. The sets of SNPs based on de novo assembled transcripts could overlap with reference-based transcripts.

To estimate the population structure of the 114 *Ae. umbellulata* accessions, Bayesian-based clustering of the biallelic SNPs was conducted using STRUCTURE (Fig. 1). L(K) and Delta K for different numbers of subpopulations presumed that the three groups (k = 3) were optimal (Supplementary Fig. S2). When k was two, the two groups corresponded to the geographic differences between the Middle East and Greece. The Middle Eastern and Greek groups were named the UmbL1 and UmbL2 lineages, respectively. When k was three, the UmbL1 lineage was divided into two sublineages, which were named the UmbL1e and UmbL1w lineages. In the structure analysis, Q-value represents the probability that an accessions belongs to a particular lineage/sublineage and was calculated for each linage. Q-value of an accession was greater than 0.8 at a particular lineage/sublineage, the accession was classified into this lineage/sublineage. The classification of accessions into each lineage were almost consistent between the population structure estimated from the SNP sets based on de novo assembled transcripts and reference-based transcripts (Supplementary Table S4). UmbL1w is distributed throughout Turkey and Syria, whereas UmbL1e originates from Iraq, Iran, and Azerbaijan. There were intermediate types between the UmbL1e and UmbL1w lineages, which were mainly found around western Turkey where their distributions overlapped. We used 87 accessions that were clearly sorted into UmbL1e, UmbL1w, or UmbL2 in both SNP sets for the downstream analyses (Supplemental Table S4). *F*_ST_, an indicator of genetic divergence between populations, was estimated to be 0.219 between UmbL1w and UmbL1e, 0.379 between UmbL1w and UmbL2, and 0.472 between UmbL1e and UmbL2. UmbL1e had more genetic divergence from UmbL2. The genetic divergence among UmbL1e, UmbL1w, and UmbL2 reflected the geographic distribution of *Ae. umbellulata* accessions belonging to each lineage.

### Characterization of phenotypic variations in *Ae. umbellulata* based on principle component analysis

**Figure 2:**
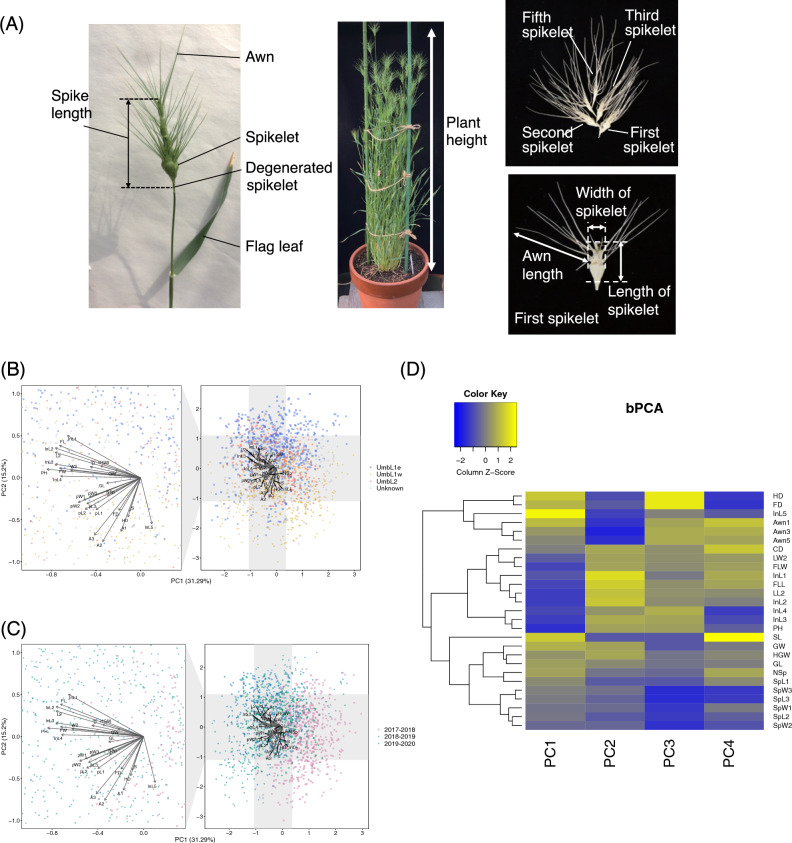
Phenotypic traits different between UmbL1 and UmbL2 lineages in *Aegilops umbellulata*. (**A**) Morphological traits measured in this study. (**B**) The PCA biplot of all 27 traits data in the *Ae. umbellulata* population shows the factor loading of each variable (gray arrows) and the scores of each accession (points) based on the first (PC1) and second (PC2) components. The raw value of traits from 114 accessions measured in the 2017–2018, 2018–2019, and 2019–2020 seasons were used and implemented by Bayesian linear model to calculate PCs. Blue squares, yellow diamonds, red circles, and gray triangles indicate UmbL1e, UmbL1w, UmbL2, and unknown lineages, respectively. (**C**) A heatmap with clustering of the Pearson correlation coefficient (*r*) between first to fourth PCs and phenotypic traits.

To investigate the phenotypic diversity of the 114 *Ae. umbellulata*, we characterized 27 *Ae. umbellulata* traits, which were evaluated in Kobe, Japan under field conditions over three or four seasons: 2016–2017, 2017–2018, 2018–2019, and 2019–2020 (Fig. 2A and Supplementary Table S5). The PCA analysis was conducted to characterize phenotypic variations in the 27 traits of *Ae. umbellulata* used in this study (Fig. 2B and Supplementary Tables S6, S7). The contributions of the first four principal components, PC1, PC2, PC3 and PC4 were 31.29%, 15.2%, 11.3%, and 6.1% respectively. Together, PC1 and PC2 explained 46.49% of the phenotypic variations. Plant height, length of first, second, third, and forth internodes, and length and width of flag leaf showed negative correlation with PC1 (Supplementary Table S6). Days to flowering and heading, awn length, spike length, and length of fifth internode exhibited negative correlations with PC2 (Supplementary Table S6). The variations of PC1 and PC2 corresponded to the seasonal differences and the lineage differentiations between UmbL1e and UmbL1w (Fig. 2B and C).

To select representatives from the traits of flowering time (Days to heading and flowering), plant height (plant height, first, second, third, fourth, and fifth internodes), spikelet size (first, second and third spikelets), and awn length (awn length of first, third and fifth spikelets), and leaf size (flag and second leaves), hierarchical clustering was performed based on the factor loadings of the tested traits (Fig. 2D). Days to flowering and heading were classified into the same group. Days to flowering were chosen as the representative of flowering time. Since awn length of first, third, and fifth spikelets were grouped together, awn length of third spikelets was used as the representative of awn length. The traits related to plant height were classified into three groups. One group includes plant height, and length of third and fifth internodes. Second group includes first and second internodes. Third group includes fifth internodes. The length of first, third and fifth internodes was used as the representatives of the traits related to plant height. The length of flag and second leaves was classified into the same cluster. The width of flag and second leaves was also categorized into the same cluster. The length and width of flag leaves were selected as the representatives of leaf size. The length of second and third spikelets were categorized to the same cluster, whereas the length of first spikelet was separated. For the evaluation of length of spikelets, the length of first and second spikelets was used. Since the width of first, second, and third spikelets were grouped together, the width of first spikelets was chosen.

### Differences in traits among UmbL1e, UmbL1w, and UmbL2 in *Ae. umbellulata*

**Figure 3:**
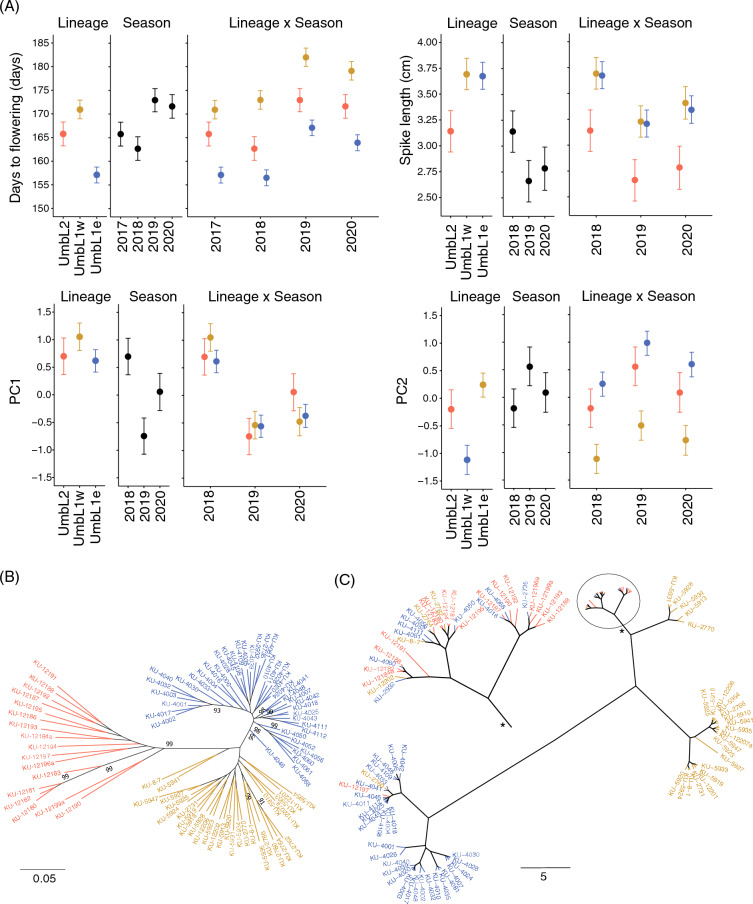
Phenotypic traits different between UmbL1e and UmbL1w lineages in *Aegilops umbellulata*. (**A**) Effects of the lineage differentiations, the seasonal differences and the interaction between and UmbL1e, UmbL1w and UmbL2 lineages, the seasons, and the interaction between lineages and seasons on the phenotypic trait variations in *Ae. umbellulata*. Posterior distributions of mean for the traits were shown. Red and blue, yellow posterior distributions indicate mean values of UmbL2, UmbL1e and UmbL1w, respectively. The center point and thin line above/below the posterior distribution designate mean and 95% credible intervals, respectively. (**B**) A Maximum Likelihood (ML) phylogenetic tree of the 87 *Ae. umbllulata* accessions. The number on the branches of the phylogenetic tree shows bootstrap probability with over 90%. (**C**) A flowering dendrogram of 87 accessions of *Ae. umbellulata* based on measurements for each season from 2017 to 2020. After normalizing days to flowering for each of the seasons 2017–2017, 2017–2018, 2018–2019, and 2019–2020, Euclidean distances were calculated. Hierarchical clustering with Ward method was carried out based on the Euclidean distance matrix. In all figures, the accessions are color-coded according to their lineages, UmbL1e, UmbL1w, and UmbL2 with blue, yellow, red. The color of external branches in the ML tree and dendrogram follows the aforementioned classification.

The effects of lineage differences, seasonal differences, and interactions between lineage and seasonal differences on the phenotypic diversity of *Ae. umbellulata* were evaluated using Bayesian generalized linear multivariate multilevel models (Bayesian GLMM). Significant differences between UmbL2 and UmbL1 were observed in the spike length, seed length, diameter of culm and width of spikelets (Fig. 3A, Supplementary Fig. S3, and Table S8). The spike length of UmbL2 was shorter than that of UmbL1e and UmbL1w. The widths of the first spikelets were greater in UmbL2 than in UmbL1e and UmbL1w. The number of spikelets per spike of UmbL2 was the lowest, while UmbL1w had the largest number of spikelets per spike. This result indicated that UmbL2 had sparse spikelets larger than the other two lineages. In contrast to the spikelets, the seeds of UmbL2 were the shortest among the lineages. Seasonal differences in these traits were detected. The interactions between lineages and seasons masked or reduced the effects of lineage differences on culm diameter, seed length, and spikelet width. Conversely, differences in spike length between UmbL1 and UmbL2 were largely independent of seasonal changes. Flowering occurred in the order UmbL1e, UmbL2, and UmbL1w, with a difference of 10 days between UmbL1e and UmbL1w (Fig. 3A and Supplementary Fig. S3 and Table S8). We also observed seasonal differences and the interaction between lineages and seasons with respect to days to flowering. The differences in days to flowering between UmbL1e and UmbL1w were greater in the 2017–2018 season compared to the 2016–2017 season. In the 2016–2017 season, the days to flowering of UmbL2 were similar to those of UmbL1w, while in the 2017–2018 season, they were closer to those of UmbL1e. The order of days to flowering among UmbL1e, UmbL1w, and UmbL2 remained stable.

Differences in internode length were observed between UmbL1e and UmbL1w (Supplementary Fig. S3). The fifth internode of UmbL1e was the shortest among all the lineages, whereas the first internode of UmbL1e were the longest. Conversely, the fifth internode of UmbL1w was the longest among all lineages, whereas the first internode of UmbL1w was the shortest. The differences in flowering time between UmbL1w and UmbL1e were positively and negatively associated with those in fifth and first internode length between them, respectively (Fig. 3 and Supplementary Fig. S3). The length of first internode increased in the season in which the days to flowering was delayed, while the length of fifth internode decreased. In contrast to days to flowering, internode length was largely influenced by the interaction between lineages and seasons. This is particularly noticeable in UmbL2, which had the longest fifth internode in the 2018–2019 season and the shortest first internode in the 2019–2020 season. The order in which the first internode of UmbL1e was longer than that of UmbL1w remained unchanged among the seasons.

The flag leaf length of UmbL1w was shorter than that of the other two lineages (Supplementary Fig. S3). The flag leaf length showed significant seasonal differences, and the interaction between lineages and seasons affected differences in flag leaf length. The shorter flag leaf of UmbL1w compared to UmbL1e was robust among the seasons. The lengths of the awns also differed among the lineages, with UmbL1w being longer than UmbL1e. Awn length remained constant over the three seasons, suggesting that these traits of *Ae. umbellulata* were less affected by environmental changes. The length of second spikelet, the width of first spikelet, seed width and 100-grain weight, no clear difference among UmbL1e, UmbL1w and UmbL2 was detected (Supplementary Fig. S3).

Principle component scores of PC1, PC2, PC3, and PC4 of 87 accessions of *Ae. umbellulata* excluding the intermediate lineages were also evaluated as phenotypic values of Bayesian GLMM (Fig. 3A). PC1 scores showed differences between UmbL1e and UmbL1w as well as among the seasons. However, the interaction between lineages and seasons masked the difference between UmbL1e and UmbL1w. On the other hand, clear differences among UmbL1e, UmbL1w, and UmbL2 were observed in the PC2 scores. Even when accounting for the interaction between lineages and seasons, the differences among them were clearly discernible. The lineage differences were also detected in the PC3 and PC4 (Supplementary Fig. S3). The difference in PC3 between UmbL1e and UmbL1w were evident, while UmbL1e and UmbL2 were distinct in PC4 even when considering the interaction between linages and seasons. These findings supported the notion that the variations of PC1 could be attributed to the seasonal differences, whereas the variations of PC2, PC3 and PC4 reflected the lineage differentiations.

### Relationship between genetic divergence and days to flowering in *Ae. umbellulata*

To examine the correlation between genetic distance and phenotypic differences in *Ae. umbellulata* accessions, a phylogenetic tree of the 87 accessions of *Ae. umbellulata* was constructed using the maximum likelihood (ML) method (Fig. 3B). Hierarchical clustering analysis of days to flowering in the four seasons was conducted for these 87 accessions (Fig. 3C). As expected, the phylogenetic tree also supported two distinct lineages, UmbL1 and UmbL2, which were supported by high bootstrap probabilities. The two sublineages, UmbL1e and UmbL1w, were clearly separated. The external branch length of UmbL1 was shorter than that of UmbL2, implying a recent expansion of UmbL1 into its habitats in Turkey, Iraq, and Iran. KU-8-7 and KU-5941 were distinct from the other accessions of UmbL1w, but their positions were not strongly supported by high bootstrap probability.

In the hierarchical clustering of days to flowering, most accessions of UmbL1e were distinct from those of UmbL2 and UmbL1w (Fig. 3B). The accessions of UmbL1w and UmbL2 were close but separated. These results corresponded to the difference inferred using the Bayesian GLMM (Fig. 3A). A comparison of the phylogenetic tree and hierarchical clustering showed that the genetically divergent lineages UmbL1w and UmbL2 were closer than the genetically similar lineages UmbL1w and UmbL1e in the relationship of *Ae. umbellulata* accessions based on days to flowering.

### Correlation between latitude/longitude, elevation, climate, and traits

**Figure 4:**
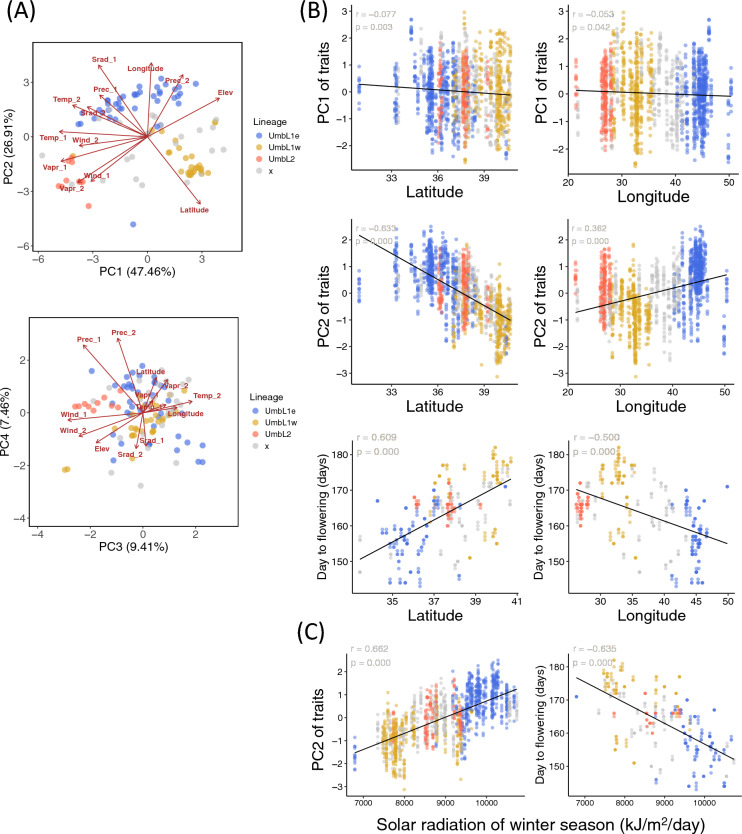
Relationship between elevation, climates, latitude/longitude, and phenotypic divergence between the lineages. (**A**) The PCA biplots of elevation and 10 sets of climate data in the 114 *Ae. umbellulata* accessions shows the factor loadings of each variable (red arrows) and scores of each accession (points) based on the first (PC1), second (PC2), third (PC3), fourth (PC4) components. The analysis employed five monthly climatic data based on WorldClim version 2.1 (Temp: mean temperature, Prec: mean precipitation, Srad: solar radiation, Vapr: water vapor pressure, Wind: wind speed) along with elevation data (Elev), and they were categorized into two seasons: winter season (November to February, labeled "_1") and summer season (March to June, labeled "_2"). Axis labels display the percentage contribution. (**B**) Scatter plots showing relationship between the PCA scores (PC1 and PC2) obtained from the PCA conducted on the traits (Fig. 2) and day to flowering of the 114 accessions of *Ae. umbellulata* with respect to latitude/longitude of their habitats. (**C**) Scatter plots showing relationship between the PC2 scores obtained from the PCA conducted on the traits (Fig. 2) and day to flowering of the 114 accessions of *Ae. umbellulata* with respect to Solar radiation of winter season (Srad_1). In figures (**B**) and (**C**), the day to flowering values in the 2017–2018 season were utilized. A linear regression line, correlation coefficient (*r*), and p-value of correlation test are shown. In all plots, the colors represent UmbL1e, UmbL1w, UmbL2, and unknown lineages, with blue, yellow, red and gray.

To characterize the habitat environments of the 114 accessions of *Ae. umbellulata*, PC analysis was performed using longitude, latitude, elevation and mean values of monthly climate data in the winter season from November to February and in the summer season from March to Jun (Fig. 4). The climate data include temperature, precipitation, solar radiation, water vapor pressure, and wind speed. The contributions of the first to four principal components, PC1, PC2, PC3 and PC4 were 47.46%, 26.91%, 9.41% and 7.46% respectively (Fig. 4A). Temperature, water vapor pressure, and wind speed were negatively correlated with PC1 in both winter and summer seasons, while elevation and summer precipitation were positively correlated with PC1. In PC1, UmbL1w and UmbL2 were separated. UmbL2 was distributed over Greek islands with low elevation and high wind speed compared to western Turkey where UmbL1w was distributed. Summer and winter solar radiation and precipitation were positively correlated with PC2. UmbL1e was separated in UmbL1w and UmbL2 in PC2. Both latitude and longitude clines were observed in PC2. In particular, the latitude was consistent with the exact opposite direction of the factor loadings of winter solar radiation and precipitation. In PC3 and PC4 plot, the direction of factor loadings of longitude was identical or exact opposite with that of temperature, wind speed and elevation. The variations of PC3 were associated with differences between UmbL1 and UmbL2.

We examined whether latitudinal or longitudinal clines was detected in the principal component scores of PC1 and PC2 for the traits, and in the four traits (days to flowering, flag leaf length, awn length, first internode length) that showed differences between UmbL1e and UmbL1w even when considering the interaction between lineages and seasons. Principal component scores of PC1 for the traits were not correlated with latitude and longitude, whereas principal component scores of PC2 for the traits were correlated with latitude and longitude (Fig. 4B). In particular, the correlation between the scores of PC2 and latitude was apparent. Days to flowering exhibited a negative correlation with longitude, while showing a significant positive correlation with latitude (Fig. 4B). Given that solar radiation was significantly correlated with latitude and is a factor of regulating flowering time, we assumed that latitudinal clines with gradients of solar radiation have the stronger effect on days to flowering in *Ae. umbellulata* (Fig. 4C).

Awn length were positively correlated with latitude and negatively correlated with longitude. The first internode length, and flag leaf length showed a negative correlation with latitude and a weakly positive correlation with longitude (Supplementary Fig. S4). These correlations were more evident in 2018. Differences in these morphological traits were continuous across latitudes, suggesting weak latitudinal clines.

## Discussion

The tested accessions of *Ae. umbellulata* diverged into two distinct lineages: UmbL1 and UmbL2. UmbL2 and UmbL1 were geographically and phenotypically distinct. UmbL2 is limited to Greece and western Turkey, whereas UmbL1 is widely distributed from central Turkey to northern Iraq. UmbL2 had shorter spikes, fewer spikelets, and longer seeds than UmbL1. However, the 100-grain weight did not differ between lineages. This suggests that UmbL1 and UmbL2 have different reproductive investment strategies. In general, a negative correlation between grain size and grain number exists^31^. Plants with smaller seeds produce more of them. Plants with larger seeds are superior to those with smaller seeds in terms of seed viability, germination, and early elongation after germination, but in terms of habitat expansion, species that produce many small seeds are more successful than species that produce fewer, large seeds^32^. The short internal and external branches of UmbL1 in the phylogenetic tree suggested the more recent expansion of UmbL1 than UmbL2 (Fig. 3B). This difference in reproductive investment strategies between the lineages may be related to the fact that UmbL1 has a larger distribution area than UmbL2. In *Aegilops markgrafii* (Greuter) Hammer, there is a geographic border between West Anatolia and Central Anatolia in Turkey based on intraspecific hybrid sterility^33^. However, the border of the habitats of UmbL1 and UmbL2 appears to be further west than that in *Ae. markgrafii*.

UmbL1 is further divided into two sublineages, UmbL1e and UmbL1w. Although UmbL1e and UmbL1w are genetically and geographically close, they are phenotypically divergent. The phenotype of UmbL1w was closer to that of UmbL2 than to that of UmbL1e. UmbL1e exhibited the earliest flowering, followed by UmbL2 and then UmbL1w. The disparity between UmbL1e and UmbL1w was more significant than that between UmbL1e and UmbL2, as was the difference in flag leaf length and first internode length. The divergence of phenotypic traits did not correlate with genetic divergence between lineages/sublineages in *Ae. umbellulata*. This discrepancy can be explained by the fact that latitudinal differences among habitats determine phenotypic divergence among *Ae. umbellulata* lineages more strongly than lineage differentiations. Although trait variations were clearly linked to both the latitude and longitude of their habitats (Fig. 1), latitudinal differences showed a stronger correlation with traits than longitudinal differences (Fig. 4).

Significant latitudinal clines in the days to flowering were detected in *Ae. umbellulata* in the same environment (Fig. 4), suggesting latitudinal clines during flowering time in *Ae. umbellulata* were genetically determined. Kobe, Japan (34.6901° N) corresponds to Tikrit in Iraq and is close to the latitude of the UmbL1e habitat. The flowering time of UmbL1w was predicted to be later than that of UmbL1e if UmbL1w were transplanted into the habitats of UmbL1e. Diversification in the timing of flowering serves as a mechanism for pre-mating isolation as it reduces the likelihood of mating between plants with different flowering times, thereby restricting gene flow between them^1–4^. Considering *Ae. umbellulata* is a selfing species with low outcrossing, in addition to differences in flowering time, which potentially promotes lineage differentiation in *Ae. umbellulata*. If the gene flow is restricted, other traits are also expected to be differentiated. In *Ae umbellulata*, there were both negative and positive correlations between days to flowering and morphological trait values (Fig. 4). UmbL1e with early flowering had longer flag and first internodes, and shorter awn length and 5th internode than UmbL1w with late flowering. However, the differences in days to flowering between UmbL1e and UmbL1w were continuous rather than discrete. An intermediate lineage between UmbL1e and UmbL1w exists in the area of their overlapping distributions. These observations suggest that pre-mating isolation between these two sublineages in the overlapping region is incomplete. The effects of differences in flowering time on sublineage differentiation may be partial.

The latitudinal cline of flowering time is considered evidence of strong natural selection for adaptation to local environments. The causal genes of flowering time could be targets for natural selection. In the related species of *Ae. umbellulata*, candidate genetic factors formed latitudinal clines have been discussed. *Ppd* genes involved in photoperiod sensitivity are candidate genes that explain the latitudinal cline during flowering in *Ae. tauschii* and common wheat^13,14^. The photoperiod-insensitive allele of *Ppd* allows photoperiod-independent flowering and facilitates early flowering. The GWAS of the 242 accessions of *Ae. tauschii* identified a significant peak on chromosome 7D, coinciding with the location of *FT* gene^34^. Additionally, QTL analyses conducted with Recombinant Inbred Lines (RILs) derived from the F_1_ cross between two divergent lineages, TauL1 and TauL2, also detected significant QTL around the *FT* gene^35^. These studies indicate that *FT* is a major cause of variation in flowering time in *Ae. tauschii*. However, the association between *FT* and the latitudinal cline of flowering time remains unclear. These reports collectively underscore that latitudinal clines can be explained by the vernalization pathway, the photoperiod pathway, or florigen itself.

Diversification of flowering has also been observed in *T. monococcum* ssp. *aegilopoides*, which has several lineages. The lineage distributed in western Turkey and Greece exhibits late flowering, whereas another lineage in eastern Turkey and Iraq exhibits early flowering^36^. A latitudinal cline in flowering time in *T. monococcum* ssp. *aegilopoides* was also detected (Supplementary Fig. S5). Accessions originating from lower latitudes showed early flowering, and accessions from upper latitudes showed late flowering. The habitat of *T. monococcum* ssp. *aegilopoides* overlapped with that of *Ae. umbellulata*, it is likely that directional selection affected the flowering time to adapt to the local environment. This similar tendency of flowering times in both species suggests that the same flowering control pathway is responsible for variations in the flowering times of both species. Further studies are needed to determine whether latitudinal clines affecting the flowering time in *Ae. umbellulata* and *T. monococcum* ssp. *aegilopoides* are caused by polymorphisms in genes in the vernalization pathway or in the photoperiodic pathway.

Temperature and precipitation also differ between the UmbL1e and UmbL1w habitats (Supplementary Fig. S6). The habitat temperature of UmbL1w in winter season was lower than that of UmbL1e. In the UmbL1e habitat, drying was severe in early summer. Therefore, temperature and precipitation in local environments can promote divergence in flowering time between sublineages in addition to latitudinal differences.

We found that the divergence of phenotypic traits did not correlate with genetic divergence or geographic distance. One of the hypothetical mechanisms explaining this discrepancy is divergent ecological selection^2^. Even when two populations are geographically close, if environments between these populations are distinct, divergence of traits could be preceded by divergent selection, resulting in reproductive isolation. The observed differences in flowering time between *Ae. umbellulata* could cause pre-mating reproductive isolation in plants. Recently, chromosome-scale genome sequences of *Ae. umbellulata* have also been published^29^. This genomic information is useful for identifying causal genes contributing to lineage differentiation. The next question to address is whether post-mating reproductive isolation occurs between these sublineages. To confirm the extent of post-mating reproduction between UmbL1e and UmbL1w, comprehensive surveys of the crossability and fertility of hybrids between these sublineages are necessary.

## Methods

### Plant materials

A total of 114 accessions of *Ae. umbellulata* were randomly selected from the seed stocks of the National BioResource Project-Wheat, Japan. The selected accessions included 19 Greek, 53 Turkish, 34 Iraqi, four Iranian, two Syrian, one Azerbaijani, and one Cypriot accession (Fig. 1 and Supplementary Table S1). To extract RNA for RNA sequencing, *Ae. umbellulata* were grown in jiffy pots under 24 °C, 16 h light, and 8 h dark conditions.

### Construction of reference transcripts of *Ae. umbellulate* for SNP calling

The de novo assembled transcripts that we had previously developed using Trinity software with default options^37^ from 300 bp paired-end reads^27^ were used as references for the alignment of short reads. In addition, transcripts of the *T. aestivum* cv. Chinese Spring D-genome is supported by deep RNA sequencing data from multiple tissues and growth stages, providing high-confidence transcript sequences^28,38^. The U-genome of *Ae. umbellulata* was genetically similar to the D-genome of *Ae. tauschii* and *T. aestivum*^39,40^. For these reasons, we reconstructed *Ae. umbellulata* transcripts by aligning RNA-seq reads from *Ae. umbellulata* to high-confidence transcripts in the CS D-genome. We used 300 bp paired-end reads of RNA-seq for mature leaves of *Ae. umbellulata* KU-4017 (DRA006404), which was used for de novo transcriptome assembly^27^. Quality control of RNA-seq reads was performed using Trimmomatic version 0.33^41^ to remove adapter sequences with an average Phred quality score per 4 bp of less than 30 bp and a length of less than 100 bp. The filtered reads were aligned to the transcripts of the CS D-genome using BWA version 0.7.17^42^. SNP and indel calling were performed using VarScan version 2.4.3^43^. When SNP sites had less than 10 coverage of reads and had less than 95% of alternative nucleotides that were different from the nucleotides of the CS D-genome, these SNP sites were discarded. Nucleotides of the CS D-genome transcripts replaced those of *Ae. umbellulata* based on qualified SNPs using javascript vcf2diplioid^44^. To improve the quality of the predicted *Ae. umbellulata* transcripts, the process of SNP calling and replacement as described above was repeated. When the coding sequences of a species are estimated based on Illumina short reads from related species, the repetition of SNP calling and replacement processes can improve the quality of coding sequences of the focal plant species^30^. The alignment and replacement processes were repeated 16 times.

### RNA sequencing

Extraction and construction of the 3′-end mRNA-seq library were performed following the modified protocol of BrAD-seq^45^. After secondary leaves of *Ae. umbellulata* emerged, seedlings were collected and immediately frozen in liquid nitrogen. The frozen seedlings were crushed using SH-48 (KURABO, Osaka, Japan) and placed in Lysis Binding Buffer (LBB) to disable mRNA degradation. mRNA was extracted from crushed leaves in LBB using Oligo d(T)25 Magnetic Beads (New England BioLabs, Ipswich, MA, USA). To fragment the mRNA, it was incubated for 3 min in buffer. Except for size selection of the library after enrichment PCR, the remaining processes in the library construction were performed according to the BrAD-seq protocol^45^. To select a library whose size covered 200–400 bp, AMPure beads (Beckman Coulter, Brea, CA, USA) were used. Adjustment of the PEG concentration in the AMPure beads allows control of the library size^46^. To filter out libraries longer than 400 bp, 42 µL of nuclease free water and 27.5 µL of twofold AMPure XP beads in 20% PEG ABR solution were added to 8 µL of enriched PCR products. The mixture was incubated for 5 min at room temperature and then placed on a magnetic tray for 5 min. After the supernatant was transferred to fresh tubes, 12.6 µL of two-fold AMPure XP beads in 20% PEG ABR was added to the supernatant to remove libraries shorter than 200 bp. The mixture was incubated for 5 min and placed on a magnetic tray for 5 min, after which the supernatant was discarded. Pellets were washed twice with 260 µl 80% ethanol on the magnetic tray. After discarding residual ethanol, the pellets were air-dried for 5 min. The libraries were eluted by 11 µL of 10 mM Tris–HCl buffer pH 8.0. After adjusting the concentration of each library to 6 nM, the libraries were mixed and sequenced with 150-bp paired-end reads on an Illumina HiSeq series.

### Quality control, alignments and SNP calling for the 114 *Ae. umbellulata*

Because the reverse reads contained poly A tails of mRNA, only 150 bp forward reads were used for SNP calling. By using the software Trimmomatic version 0.33^41^, adapter sequences were removed from forward reads, and then qualified reads, of which average quality per 4 bp and length were ≥ 20 and > 75 bp (“trimmomatic SE -phred33 -threads 32 sample1_rawread.fastq.gz sample1_clean.fastq.gz ILLUMINACLIP:TruSeq2-SE.fa:2:30:10 SLIDINGWINDOW:4:20 MINLEN:75”), were obtained. Qualified reads were aligned to the deduced reference transcripts using BWA version 0.7.17^42^ ("bwa mem -t 32 reference.fasta sample1_clean.fastq.gz | samtools view -SbF4- | samtools sort -@ 32 -o sample1.bam”). SAMtools^47^ and VarScan version 2.4.3^43^ were used for SNP calling (“samtools mpileup -f reference.fasta_sample1.bam > sample1.mpileup”, and then “varscan mpileup2snp sample1.mpileup -min-coverage 10 -min-var-freq 0.9 -min-freq-for-hom 0.9 -p-value 0.05 -output-vcf 1 -variants > sample1_snp.vcf”). A position where the coverage of reads was five or more and over 80% of reads designated a nucleotide that was different from the reference transcript one, was regarded as an SNP site. From the detected SNP sites, we extracted a set of sites where all the accessions had three or more mapped reads and did not contain any unconfirmed nucleotides and called them 100% covered SNP set. A 100% covered SNP set was used for population structure analyses. In addition, we extracted a set of SNP sites where over 95% and over 90% of accessions had confirmed nucleotides that were supported by three or more mapped reads. The sets of these SNP sites were called 95% covered SNP set and 90% covered SNP sets.

### Population structure analyses

Bayesian inference of the population structures of the 114 *Ae. umbellulata* was performed using STRUCTURE version 2.3.4^48^ and STRUCTURE Harvester^49^. The number of clusters in the ancestral population (k) was set to one, two, three, four, and five. Without providing information on the geographic origins of *Ae. umbellulata* a priori, the parameters of the population structures for each k were inferred using MCMC with 110,000 iterations containing 10,000 iterations for burn-in. Ten trials were conducted for each k, and L(K) and delta k were calculated to determine the optimal k^50^. The Q value is calculated for each k value. The results of the 10 trials were visualized as bar plots using CLUMPP version 1.1.2^51^. Lineage of accessions was determined based on the highest Q-value with over 80%. If Q-value was less than 80%, accessions were not classified into any lineage. The pie charts of Q-value of each accession on the geographic map were generated with map_data(), ggplotGrob(), and ggplot() functions under “ggplot2” package version 3.5.0^52^ in R software ver. 4.3.1. The map dataset was sourced from the “ggplot2” package.

A Maximum Likelihood (ML) phylogenetic tree of the 87 *Ae. umbellulata*, classified into the three lineages was constructed based on a set of non-redundant SNPs using the Molecular Evolutionary Genetics Analysis software (MEGA) version 7.0.26^53^. The Kimura 2-parameter model was used as the nucleotide substitution model. Bootstrap probabilities for supporting the bifurcation of branches in trees were calculated in 1000 replications. *F*_ST_ was estimated based on SNPs estimated from the reference-based transcripts by using R package “hierfstat.”

### Measurements of 29 traits in 114 *Ae. umbellulata* accessions.

To assess the phenotypic traits of *Ae. umbellulata*, two plants per accession, were cultivated in clay pots randomly placed in an unregulated temperature vinyl house at Kobe University, Nada-Ku, Kobe (N34.7°, E135.2°) from early November to early June in four seasons 2016–2017, 2017–2018, 2018–2019 and 2019–2020. For pot soil preparation, a fertilizer mixture containing 8% each of N, P, and K was applied at a rate of approximately 70 g/m^2^. A total of 27 traits were evaluated. Days to heading, days to flowering, 100-grain weight, length of grain, and width of grain were measured in all four seasons, whereas other traits were only measured in three seasons. These traits, except 100-grain weight, length of grain, and width of grain, were assessed in three or five replicates per plant. The day-to-heading and day-to-flowering periods were defined as the number of days from sowing to heading and flowering, respectively. A survey of the days to heading was conducted from 9:00 to 10:30 in the morning. The heading date was defined as the day on which the tip of the spike detached from the flag leaf. A survey of days to flowering was conducted from 9:00 to 10:30. The day on which the anthers completely emerged from the florets was defined as the flowering date. Culm length was measured from the crown to the neck of the spike. Plant height was defined as the height with the largest sum of culm and spike lengths (Fig. 2A). The culm diameter was measured immediately below the peduncle. Spikelet length and width were measured for three spikelets per spike. The length and width of grains from the second floret of each accession were measured using *SmartGrain* software version 1.2, originally developed for the high-throughput phenotyping of rice (*Oryza sativa*) seeds^54^. Grain length and width were recorded for a minimum of five seeds from each accession and line following the *SmartGrain* protocol.

Principal component analyses (PCA) of 24 traits without grain traits measured in the 2017–2018, 2018–2019 and 2019–2020 seasons were conducted using R software ver. 4.3.2 with Bayesian PCA (bpca) function under “pcaMethods” package^55^. Measured values were scaled by uv (unit variance) method. After scaling, missing values were implemented with a Bayesian model with 100 estimation steps before PCA was conducted.

Trait differentiations between lineages were tested under Bayesian GLMM using the Stan (brms) package^56^. Posterior distributions of the means of explanatory variables were obtained using the model described in the following equation to estimate the effects of lineage and seasonal differences on traits, the PCs in *Ae. umbellulata*.

$${\gamma }_{i} \sim Normal (0, { \delta }^{2})$$

$${\mu }_{i} \sim {\beta }_{0}+{\beta }_{1}{x}_{i1}+{\beta }_{2}{x}_{i2}+{\beta }_{3}{{x}_{i1}*x}_{i2}+{\gamma }_{i}$$

$${y}_{i} \sim Normal ({\mu }_{i}, { \delta }^{2})$$

Individual identification was incorporated into the model as random effect ${\gamma }_{i}$. Lineage differences, seasonal differences and their interaction were incorporated into the model as fixation effects ${\beta }_{1}$, ${\beta }_{2}$ and ${\beta }_{3}$. ${\beta }_{0}$ is the intercept of the fixed effect. Response variables ${y}_{i}$ indicate the measurements of traits. The posterior distributions of the parameters were calculated using the default priors of brms and four chains with 5000 iterations. In each chain, the first 1000 iterations were not used for estimating the posterior distributions as burn-in periods. A total of 16,000 posterior samples were obtained. By checking whether Rhat, MCMC convergence statistic, was less than 1.1 for all parameters of the models, we confirmed that all parameters were convergent. Based on the posterior distributions, the means and two-sided credible intervals of coefficients were estimated.

Clustering analysis was conducted on the flowering of 87 accessions, classified into the three lineages. After normalizing days to flowering for each of the seasons 2017–2017, 2017–2018, 2018–2019, and 2019–2020, Euclidean distances were calculated. Hierarchical clustering was carried out based on the Euclidean distance matrix. The Ward method, implemented with the R package “stats”, was utilized for clustering. The resulting dendrogram was visualized using the R packages “ape”, “ggtree”, and “dendextend”.

### Principle component analysis of climatic and elevation data in the habitat of 114 *Ae. umbellulata*

PCA was conducted on climatic and elevation data to examine the relationship between these factors and the longitude, latitude, and lineage of *Ae. umbellulata* habitats. Climate data spanning from 1970 to 2000 were obtained from the WorldClim version 2.1 (https://www.worldclim.org)^57^ database, encompassing monthly average temperature, precipitation, solar radiation, water vapor pressure and wind speed. The average values for two seasons (Winter season: November to February, and Summer season: March to June) were calculated to cover the growing season of *Ae. umbellulata*. Elevation data were obtained from SRTM elevation data (WorldClim version 2.1). The dataset was normalized and PCA was performed using the “stats” prcomp() function in R to determine the contribution ratio of each principal component (PC), PC scores for the 114 accessions, and factor loadings for climate and elevation factors. Results were visualized by using the R package “ggplot2”^52^.

### Supplementary Information

Supplementary Figures.

Supplementary Tables.

## Data Availability

All data generated or analyzed during this study are included in this published article and its Supplementary Information files and are deposited in the DDBJ database under the following accession numbers: DRA017516.

## References

[1] Ledyard Stebbins G (1950). Variation and Evolution in Plants.

[2] Nosil P (2012). Ecological Speciation.

[3] Gaudinier A, Blackman BK (2020). Evolutionary processes from the perspective of flowering time diversity. New Phytol..

[4] Orsucci M, Sicard A (2021). Flower evolution in the presence of heterospecific gene flow and its contribution to lineage divergence. J. Exp. Bot..

[5] Tyler L (2016). Population structure in the model grass is highly correlated with flowering differences across broad geographic areas. Plant Genome.

[6] Debieu M (2013). Co-variation between seed dormancy, growth rate and flowering time changes with latitude in *Arabidopsis thaliana*. PLoS ONE.

[7] Tooke F, Battey NH (2010). Temperate flowering phenology. J. Exp. Bot..

[8] Johansson M, Staiger D (2015). Time to flower: Interplay between photoperiod and the circadian clock. J. Exp. Bot..

[9] Stinchcombe JR (2004). A latitudinal cline in flowering time in *Arabidopsis thaliana* modulated by the flowering time gene *FRIGIDA*. Proc. Natl. Acad. Sci. USA.

[10] Caicedo AL, Stinchcombe JR, Olsen KM, Schmitt J, Purugganan MD (2004). Epistatic interaction between *Arabidopsis FRI* and *FLC* flowering time genes generates a latitudinal cline in a life history trait. Proc. Natl. Acad. Sci. USA.

[11] Stinchcombe JR (2005). Vernalization sensitivity in *Arabidopsis thaliana* (Brassicaceae): The effects of latitude and *FLC* variation. Am. J. Bot..

[12] Izawa T (2007). Adaptation of flowering-time by natural and artificial selection in Arabidopsis and rice. J. Exp. Bot..

[13] Matsuoka Y, Takumi S, Kawahara T (2008). Flowering time diversification and dispersal in central Eurasian wild wheat *Aegilops tauschii* Coss.: Genealogical and ecological framework. PLoS ONE.

[14] Guo Z, Song Y, Zhou R, Ren Z, Jia J (2010). Discovery, evaluation and distribution of haplotypes of the wheat *Ppd-D1* gene. New Phytol..

[15] Thornsberry JM (2001). *Dwarf8* polymorphisms associate with variation in flowering time. Nat. Genet..

[16] Andersen JR, Schrag T, Melchinger AE, Zein I, Lübberstedt T (2005). Validation of *Dwarf8* polymorphisms associated with flowering time in elite European inbred lines of maize (*Zea mays* L.). Theor. Appl. Genet..

[17] Camus-Kulandaivelu L (2006). Maize adaptation to temperate climate: Relationship between population structure and polymorphism in the *Dwarf8* gene. Genetics.

[18] Matsuoka Y, Takumi S, Kawahara T (2015). Intraspecific lineage divergence and its association with reproductive trait change during species range expansion in central Eurasian wild wheat *Aegilops tauschii* Coss. (Poaceae). BMC Evol. Biol..

[19] Mizuno N, Yamasaki M, Matsuoka Y, Kawahara T, Takumi S (2010). Population structure of wild wheat D-genome progenitor *Aegilops tauschii* Coss.: Implications for intraspecific lineage diversification and evolution of common wheat. Mol. Ecol..

[20] Schachermayr G (1994). Identification and localization of molecular markers linked to the *Lr9* leaf rust resistance gene of wheat. Theor. Appl. Genet..

[21] Chhuneja P (2008). Transfer of leaf rust and stripe rust resistance from *Aegilops umbellulata* Zhuk. to bread wheat (*Triticum aestivum* L.). Genet. Resour. Crop Evol..

[22] Edae EA, Olivera PD, Jin Y, Poland JA, Rouse MN (2016). Genotype-by-sequencing facilitates genetic mapping of a stem rust resistance locus in *Aegilops umbellulata*, a wild relative of cultivated wheat. BMC Genomics.

[23] Edae EA, Olivera PD, Jin Y, Rouse MN (2017). Genotyping-by-sequencing facilitates a high-density consensus linkage map for *Aegilops umbellulata*, a wild relative of cultivated wheat. G3.

[24] Wang J, Wang C, Zhen S, Li X, Yan Y (2018). Low-molecular-weight glutenin subunits from the 1U genome of *Aegilops umbellulata* confer superior dough rheological properties and improve breadmaking quality of bread wheat. J. Sci. Food Agric..

[25] Okada M, Yoshida K, Takumi S (2017). Hybrid incompatibilities in interspecific crosses between tetraploid wheat and its wild diploid relative *Aegilops umbellulata*. Plant Mol. Biol..

[26] Okada M (2020). Phenotypic effects of the U-genome variation in nascent synthetic hexaploids derived from interspecific crosses between durum wheat and its diploid relative *Aegilops umbellulata*. PLoS ONE.

[27] Okada M (2018). RNA-seq analysis reveals considerable genetic diversity and provides genetic markers saturating all chromosomes in the diploid wild wheat relative *Aegilops umbellulata*. BMC Plant Biol..

[28] International Wheat Genome Sequencing Consortium (IWGSC) (2018). Shifting the limits in wheat research and breeding using a fully annotated reference genome. Science.

[29] Abrouk M (2023). Chromosome-scale assembly of the wild wheat relative *Aegilops umbellulata*. Sci. Data.

[30] Paape T (2018). Patterns of polymorphism and selection in the subgenomes of the allopolyploid *Arabidopsis kamchatica*. Nat. Commun..

[31] Gupta PK, Rustgi S, Kumar N (2006). Genetic and molecular basis of grain size and grain number and its relevance to grain productivity in higher plants. Genome.

[32] Guo Q, Brown JH, Valone TJ, Kachman SD (2000). Constraints of seed size on plant distribution and abundance. Ecology.

[33] Ohta S (2000). Genetic differentiation and post-glacial establishment of the geographical distribution in *Aegilops caudata* L. Genes Genet. Syst..

[34] Gaurav K (2022). Population genomic analysis of *Aegilops tauschii* identifies targets for bread wheat improvement. Nat. Biotechnol..

[35] Miki Y (2020). GRAS-Di system facilitates high-density genetic map construction and QTL identification in recombinant inbred lines of the wheat progenitor *Aegilops tauschii*. Sci. Rep..

[36] Michikawa A (2023). Phenotypic effects of A^m^ genomes in nascent synthetic hexaploids derived from interspecific crosses between durum and wild einkorn wheat. PLoS ONE.

[37] Grabherr MG (2011). Full-length transcriptome assembly from RNA-Seq data without a reference genome. Nat. Biotechnol..

[38] Ramírez-González RH (2018). The transcriptional landscape of polyploid wheat. Science.

[39] Glémin S (2019). Pervasive hybridizations in the history of wheat relatives. Sci. Adv..

[40] Tanaka S, Yoshida K, Sato K, Takumi S (2020). Diploid genome differentiation conferred by RNA sequencing-based survey of genome-wide polymorphisms throughout homoeologous loci in *Triticum* and *Aegilops*. BMC Genomics.

[41] Bolger AM, Lohse M, Usadel B (2014). Trimmomatic: A flexible trimmer for Illumina sequence data. Bioinformatics.

[42] Li H, Durbin R (2009). Fast and accurate short read alignment with Burrows–Wheeler transform. Bioinformatics.

[43] Koboldt DC (2009). VarScan: Variant detection in massively parallel sequencing of individual and pooled samples. Bioinformatics.

[44] Rozowsky J (2011). AlleleSeq: Analysis of allele-specific expression and binding in a network framework. Mol. Syst. Biol..

[45] Townsley BT, Covington MF, Ichihashi Y, Zumstein K, Sinha NR (2015). BrAD-seq: Breath Adapter Directional sequencing: a streamlined, ultra-simple and fast library preparation protocol for strand specific mRNA library construction. Front. Plant Sci..

[46] Hosomichi K, Mitsunaga S, Nagasaki H, Inoue I (2014). A Bead-based Normalization for Uniform Sequencing depth (BeNUS) protocol for multi-samples sequencing exemplified by HLA-B. BMC Genomics.

[47] Li H (2009). The sequence alignment/map format and SAMtools. Bioinformatics.

[48] Pritchard JK, Stephens M, Donnelly P (2000). Inference of population structure using multilocus genotype data. Genetics.

[49] Earl DA, von Holdt BM (2012). STRUCTURE HARVESTER: A website and program for visualizing STRUCTURE output and implementing the Evanno method. Conserv. Genet. Resour..

[50] Evanno G, Regnaut S, Goudet J (2005). Detecting the number of clusters of individuals using the software STRUCTURE: A simulation study. Mol. Ecol..

[51] Jakobsson M, Rosenberg NA (2007). CLUMPP: A cluster matching and permutation program for dealing with label switching and multimodality in analysis of population structure. Bioinformatics.

[52] Wickham, H. *ggplot2: Elegant Graphics for Data Analysis*. ISBN 978-3-319-24277-4 (Springer, 2016).

[53] Kumar S, Stecher G, Tamura K (2016). MEGA7: Molecular evolutionary genetics analysis version 7.0 for bigger datasets. Mol. Biol. Evol..

[54] Tanabata T, Shibaya T, Hori K, Ebana K, Yano M (2012). *SmartGrain*: High-throughput phenotyping software for measuring seed shape through image analysis. Plant Physiol..

[55] Stacklies W, Redestig H, Scholz M, Walther D, Selbig J (2007). pcaMethods—a Bioconductor package providing PCA methods for incomplete data. Bioinformatics.

[56] Bürkner P-C (2017). brms: An R package for Bayesian multilevel models using Stan. J. Stat. Softw..

[57] Fick SE, Hijmans RJ (2017). WorldClim 2: New 1km spatial resolution climate surfaces for global land areas. Int. J. Climatol..

