# Supplementary material for: Discrepancy of flowering time between genetically close sublineages of Aegilops umbellulata Zhuk
Source: Sci Rep. 2024 Mar 28;14:7437. doi: 10.1038/s41598-024-57935-w (PMC10978908; doi:10.1038/s41598-024-57935-w)
Supplement: Supplementary file 1 — Supplementary Figures. [file 41598_2024_57935_MOESM1_ESM.pdf]

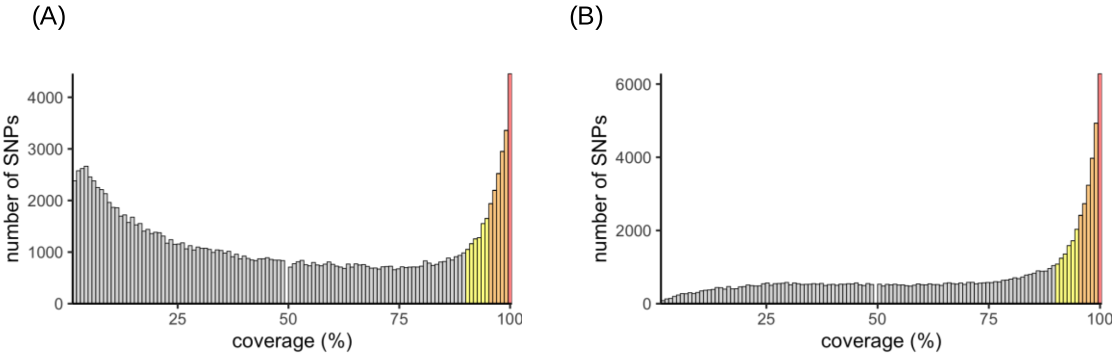

Supplementary Fig. S1 Single nucleotide polymorphisms (SNPs) among the 114 *Aegilops umbellulata* accessions in the reference-based transcript SNP set and the *de novo* transcript SNP set. Coverage of the detected SNPs among the 114 *Ae. umbellulata* accessions in the reference-based transcript SNP set (A) and the *de novo* transcript SNP set (B). Red, orange and yellow bars represent the number of SNPs which were covered by 100%, 95% and 90% of all accessions, respectively. Red, orange and yellow lines represent for the loci of SNPs which were covered by 100%, 95% and 90% of all accessions, respectively.

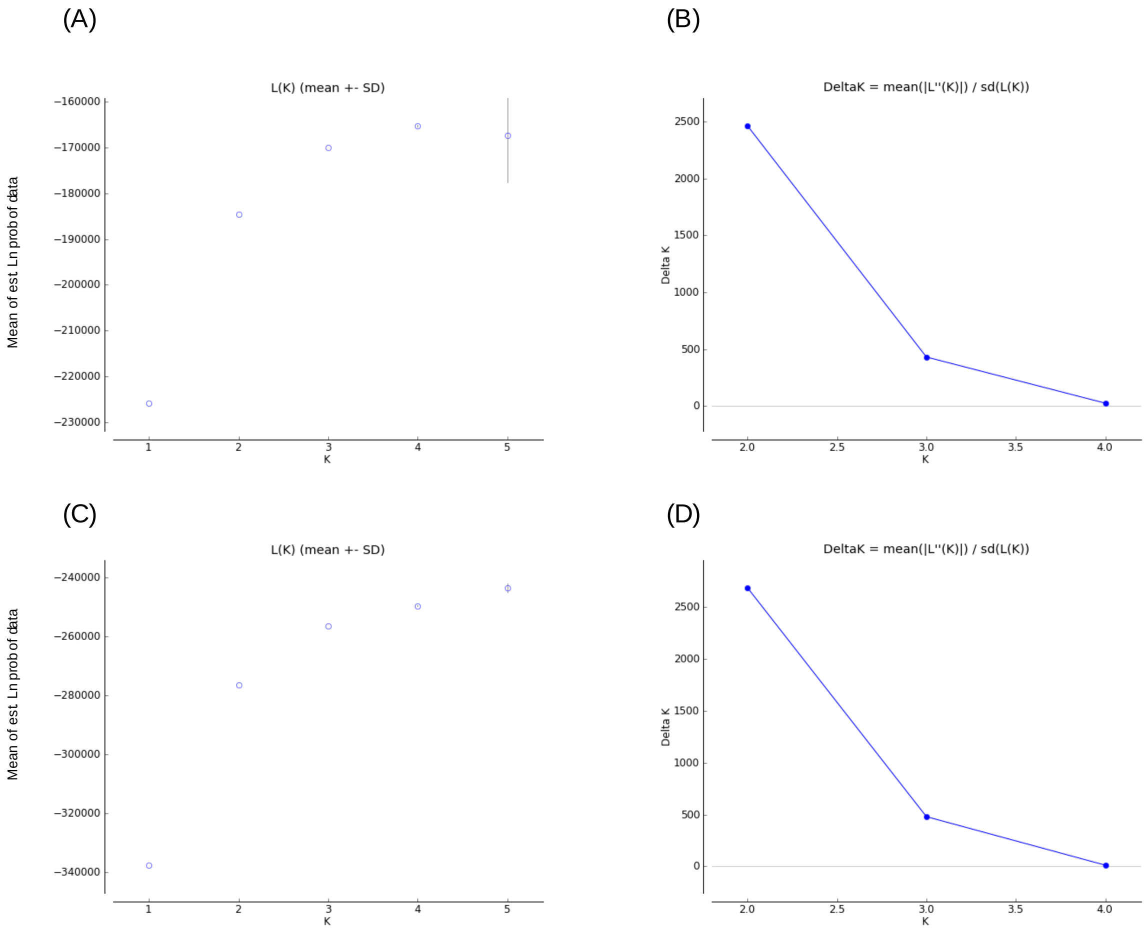

Supplementary Fig. S2 Estimation of the number of sub-populations (k) from STRUCTURE results for differing numbers of subpopulations.
(A) L(K) for differing numbers of subpopulations based on the reference-based transcript SNP set. (B) Delta (Δ)K for differing numbers of subpopulations based on the reference-based transcript SNP set. (C) L(K) for differing numbers of subpopulations based on the *de novo* transcript SNP set. (D) Delta (Δ)K for differing numbers of subpopulations based on the *de novo* transcript SNP set.

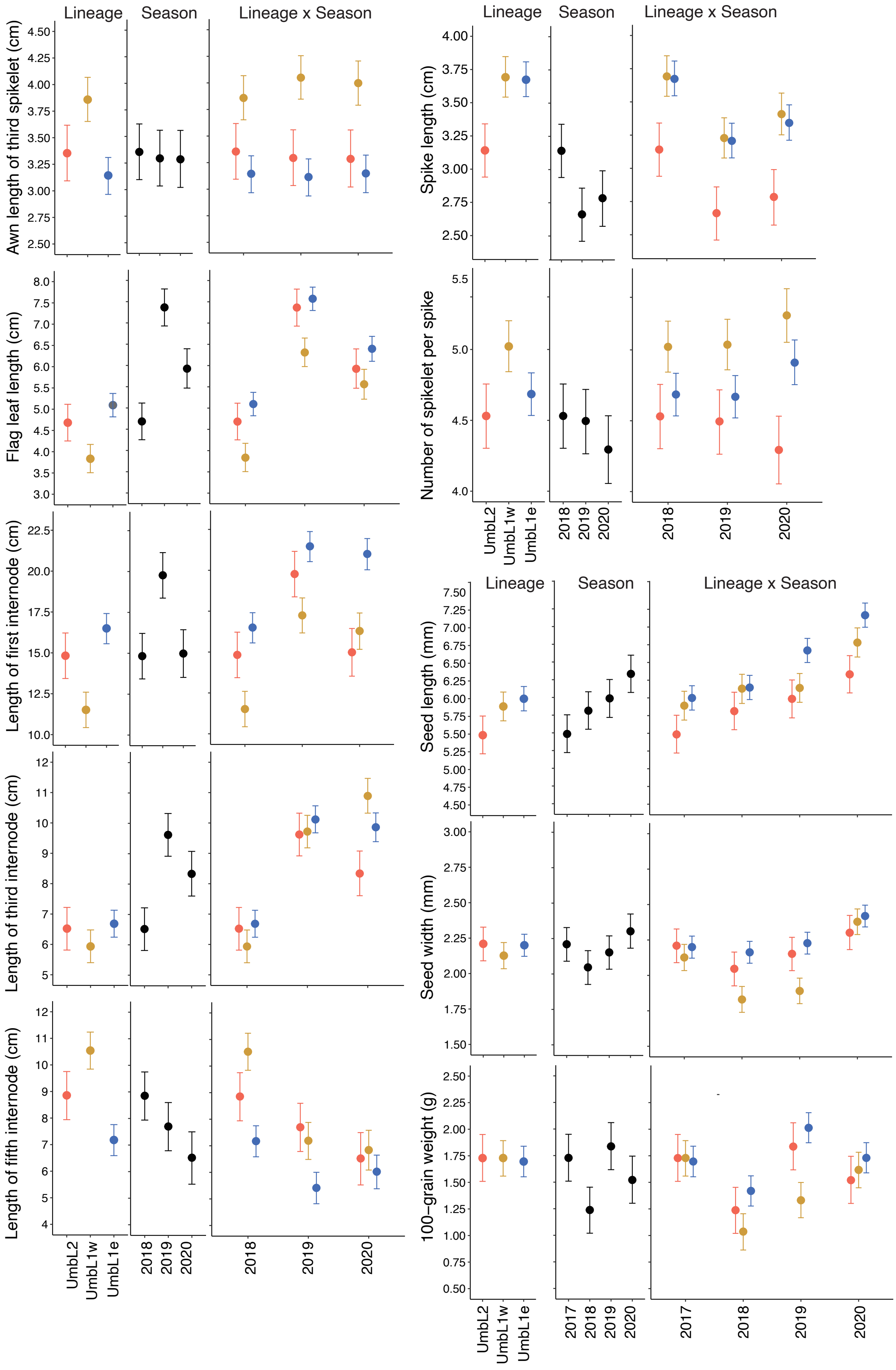

Supplementary Fig. S3 Effects of the lineage differentiations, the seasonal differences and the interaction between and UmbL1e, UmbL1w and UmbL2 lineages, the seasons, and the interaction between lineages and seasons on the phenotypic trait variations in *Aegilops umbellulata*.

Posterior distributions of means for the traits are shown. Red, yellow, and blue posterior distributions indicate mean values of UmbL2, UmbL1w, and UmbL1e, respectively. The center point and thin line above/below the posterior distribution represent mean and 95% credible intervals, respectively.

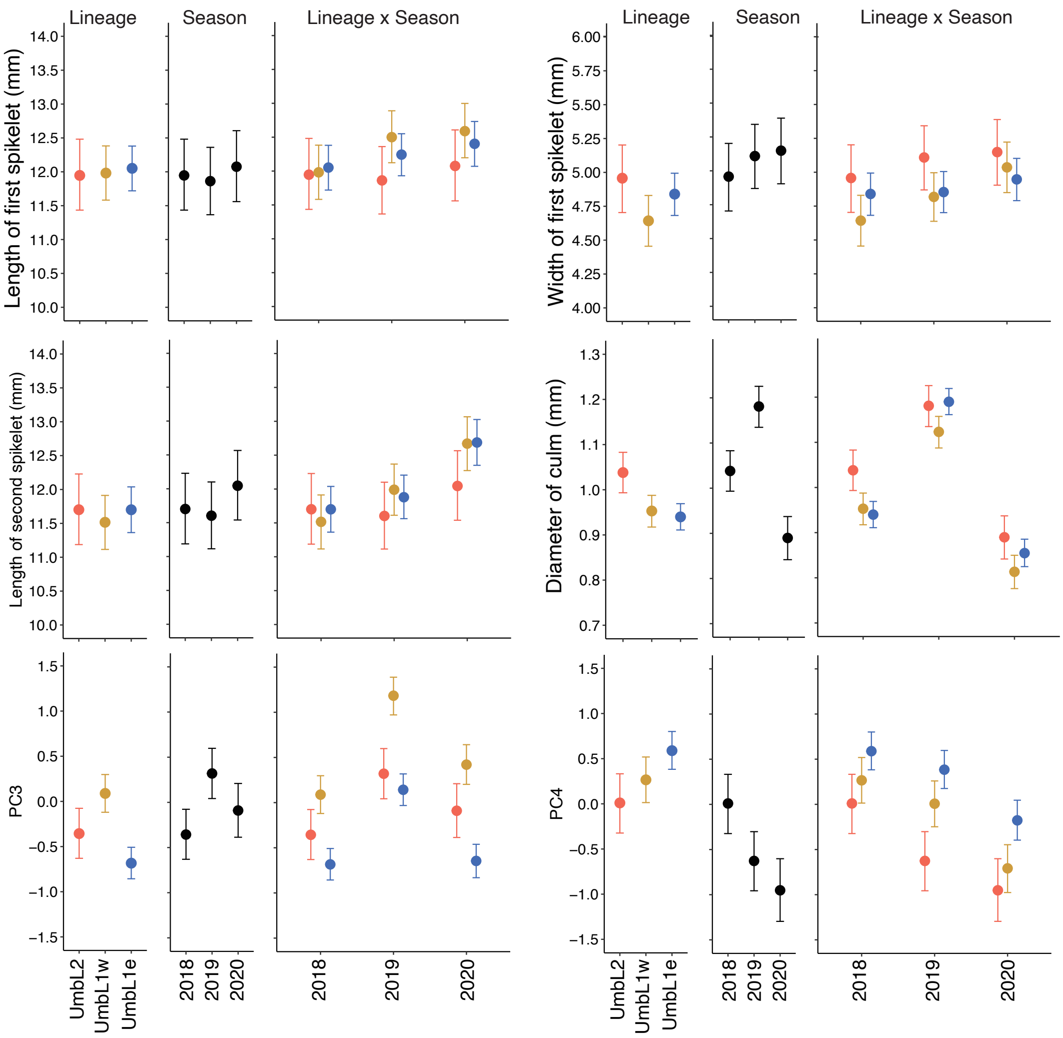

Supplementally Fig. S3 (*Continued*)

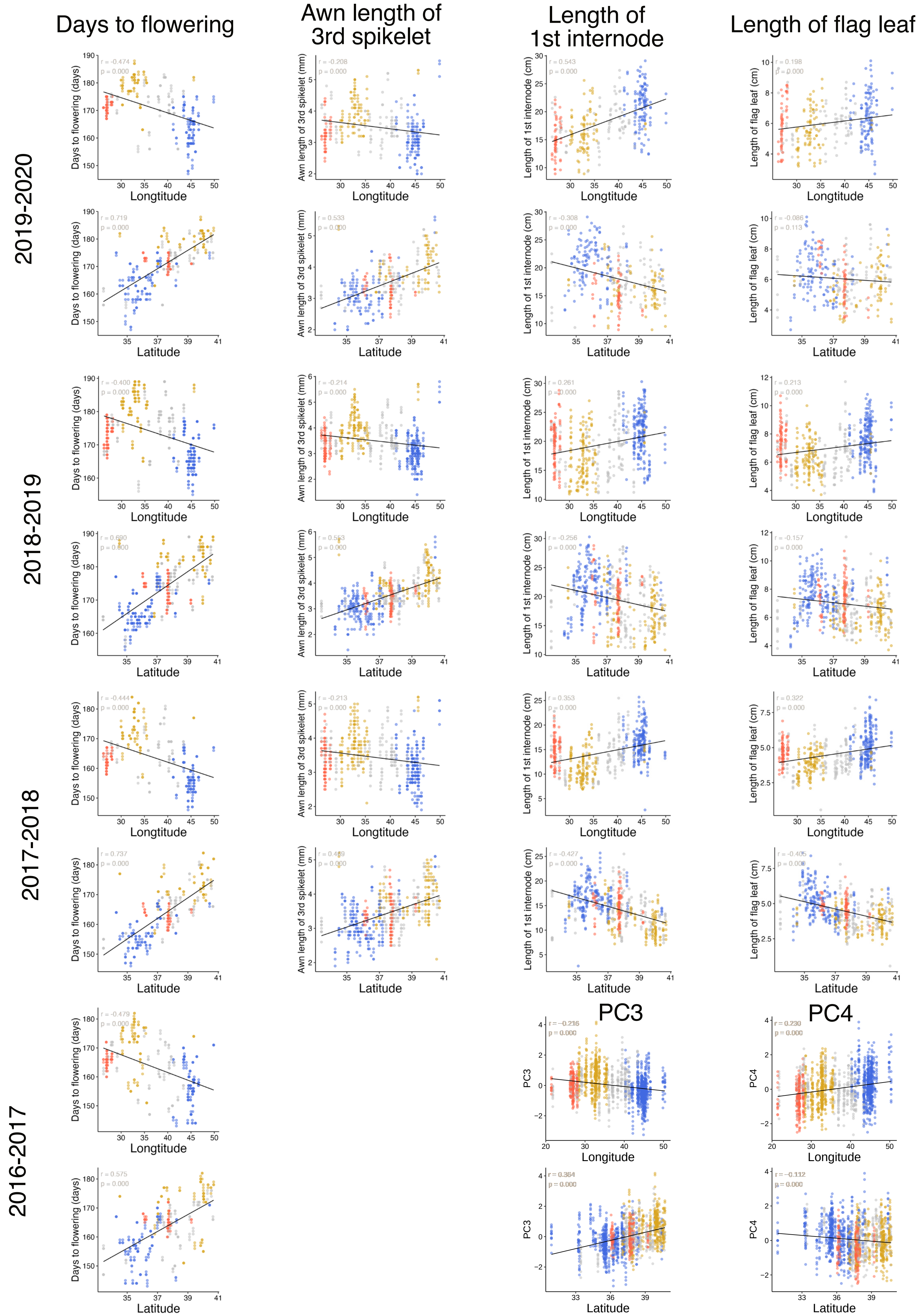

Supplementary Fig. S4 Scatter plots for the representative traits, 3rd, and 4th PC score of the 114 accessions of *Aegilops umbellulata* and longitude/ latitude of their habitats. A linear regression line, correlation coefficeint (r), and p-value of the correlation test are shown. In all plots, the colors represent UmbL1e, UmbL1w, UmbL2, and unknown lineages, with blue, yellow, red and gray.

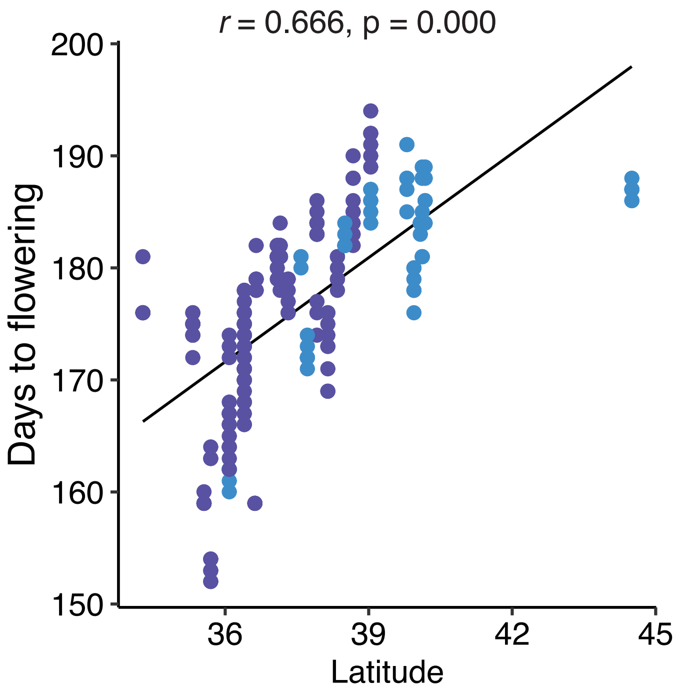

Supplementary Fig. S5 Scatter plots for the traits of *Triticum monococcum* ssp. *aegilopoides* and latitude of their habitats.

Data for days to flowering in 2017-2018 and 2018-2019 published in Michikawa et al. 2023 were used. A linear regression line, correlation coefficient (*r*), and p-value of correlation test are shown.

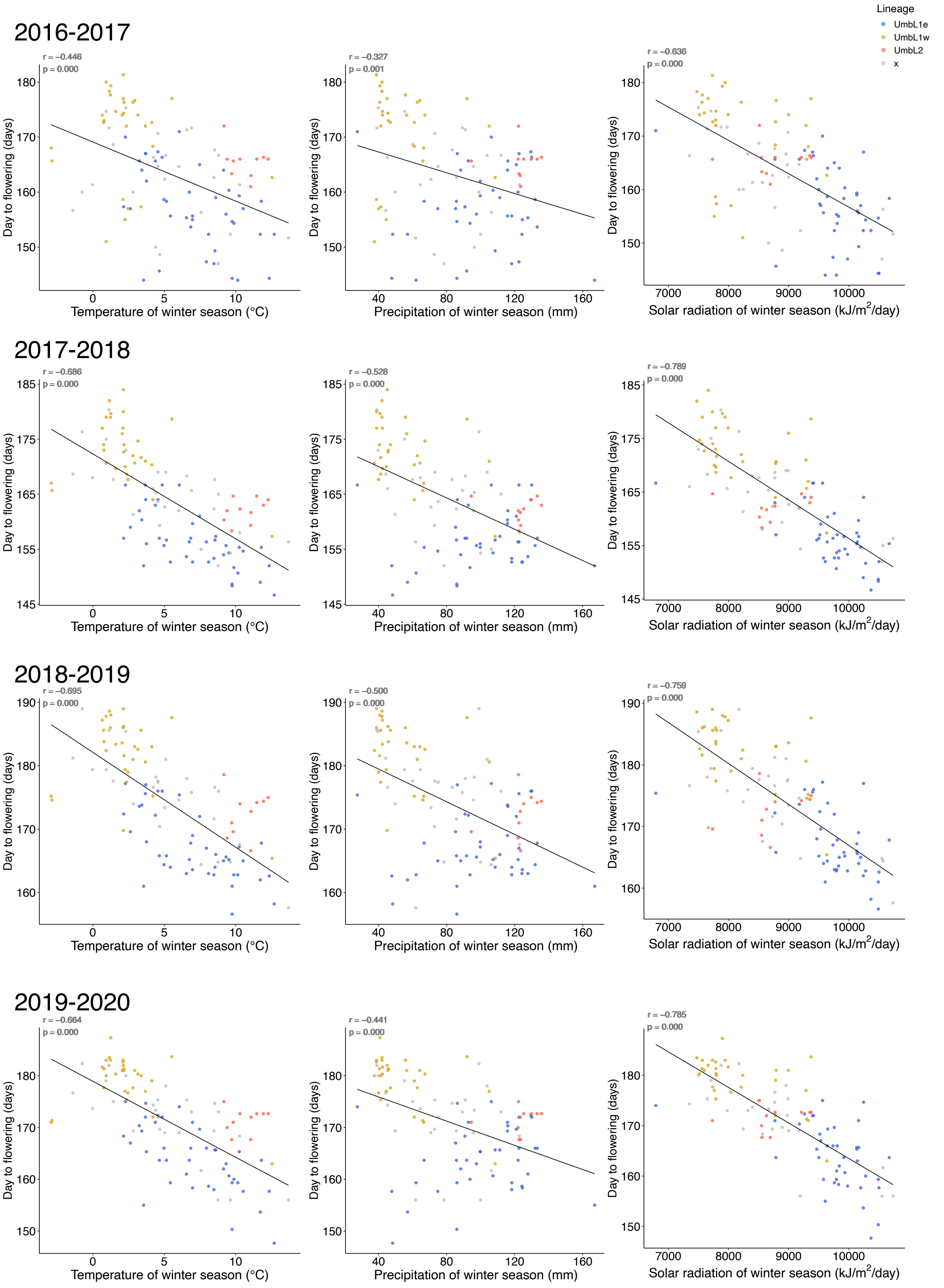

Supplementary Fig. S6 Scatter plots for the day to flowering and three climate factors (Temperature, Precipitation, and Solar radiation) in the Winter season (November to February). The day to flowering values of the 114 accessions measured in the 2017-2018, 2018-2019, and 2019-2020 seasons were utilized. A linear regression line, correlation coefficient (r), and p-value of the correlation test are shown. The colors represent UmbL1e, UmbL1w, UmbL2, and unknown lineages, with blue, yellow, red and gray.
